# Migration and Mental Health in the Aftermath of Disaster: Evidence from Mt. Merapi, Indonesia

**DOI:** 10.3390/ijerph16152726

**Published:** 2019-07-31

**Authors:** Jonathan A. Muir, Michael R. Cope, Leslie R. Angeningsih, Jorden E. Jackson, Ralph B. Brown

**Affiliations:** 1 Department of Sociology, The Ohio State University, Columbus, OH 43210, USA; 2 Department of Sociology, Brigham Young University, Provo, UT 84602, USA; 3 Institute of Community Development (APMD), Yogyakarta 55225, Indonesia

**Keywords:** environmental disasters, forced migration, internal displacement, health

## Abstract

Migration is a standard survival strategy in the context of disasters. While prior studies have examined factors associated with return migration following disasters, an area that remains relatively underexplored is whether moving home to one’s original community results in improved health and well-being compared to other options such as deciding to move on. In the present study, our objective is to explore whether return migration, compared to other migration options, results in superior improvements to mental health. We draw upon data from a cross-sectional pilot study conducted 16 months after a series of volcanic eruptions in Merapi, Indonesia. Using ordinal logistic regression, we find that compared to respondents who were still displaced (reference category), respondents who had “moved home” were proportionally more likely to report good mental health (proportional odds ratios (POR) = 2.02 [95% CI = 1.05, 3.91]) compared to average or poor mental health. Likewise, respondents who had “moved on” were proportionally more likely to report good mental health (POR = 2.64 [95% CI = 0.96, 7.77]. The results suggest that while moving home was an improvement from being displaced, it may have been better to move on, as this yielded superior associations with self-reported mental health.

## 1. Introduction

From 26 October to 5 November 2010, the region surrounding Gunung Merapi (“Mountain of Fire”)—located in Central Java, Indonesia—was devastated by a series of violent eruptions. The eruptions included repeated discharges of ash and lava and generated large eruption columns that caused pyroclastic flows to enter densely populated areas located along the slopes of the volcano. Beyond the eruptions, heavy rainfall in the area produced highly destructive lahars. Prior to the onset of these eruptions, the Indonesian government issued evacuation orders that affected 19,000 people. In total, roughly 400,000 people were displaced, 3300 homes and/or buildings were destroyed, and 383 people were killed.

In conditions such as these, disasters are often associated with forced migration [1,2,3,4,5]. Indeed, migration is a standard strategy for surviving disruptions that pose risk to both life and property [3,6,7,8]. In the case of Mt. Merapi, where a major eruption occurs approximately every 4–5 years, forced migration is quite common; yet the area surrounding the mountain remains densely populated despite the well-known dangers, as households persist in returning to their original communities once the dangers associated with an eruption have subsided [9,10,11,12]. Given that these patterns of return migration are likely associated with increased risk of future disaster events, the Indonesian disaster management agency “Badan Nasional Penanggulangan Bencana” (BNPB) conducted a risk assessment of the Mt. Merapi region and thereafter sought to provide incentives (e.g., varying forms of recovery assistance) for the resettlement of risky residential areas as a hazard mitigation strategy [10,13].

In a working paper recently presented at the International Union for the Scientific Study of Population [13,14], Muir et al. discussed findings from an impact assessment of some of these forms of recovery aid with regards to whether or not they were associated with migration decisions—particularly with regards to creating incentives for resettlement. They found that of the various forms of aid considered in their study, financial recovery aid was the only type of aid that was consistently associated with resettlement or “moving on”. However, they also found evidence suggesting that while recovery aid in the form of food and health assistance as well as remittances may not have been sufficient in and of themselves to increase resettlement, they may enhance the effect of financial recovery aid. Given the potential policy implications of these findings, and the interest in fostering resettlement among local policy makers [10], an important next step is to assess whether different migration decisions (e.g., deciding to move home or to move on) after a devastating disaster are associated with improved health and well-being, particularly in contrast to those who remain displaced. This question remains uncertain for the population at hand and, more broadly, it is a subject that has received limited theoretical and empirical attention [15,16,17].

### 1.1. Migration, Internal Displacement, and the Return Home

Migration, broadly conceptualized as a “permanent or semi-permanent change of residence” [18] (p. 49), is a fundamental component of demographic change [7,19,20]. Accordingly, it has been a critical area of focus in demographic research since Ravenstein (1885) [21] proposed his Laws of Migration. Lee (1966) refined Ravenstein’s model and therein provided a seminal contribution to the theoretical understanding of migration. Central to Lee’s theory are the roles of push and pull factors as well as intervening obstacles that prevent or delay migration.

Encapsulated within the broader examination of migration is the study of internal displacement. Here again Lee’s definition of migration as a “permanent or semi-permanent change of residence” [18] holds true (p. 49). However, more often than not, the push and pull factors influencing migration in these circumstances are unique. Internally displaced persons are often migrants who are *forced* to flee as a survival strategy against risks to life resulting from a disaster or armed conflict [3,4,5,6,7,8,15,17,22]. This contrasts with the experience of other migrants who *choose* to migrate for the purpose of maximizing life outcomes such as improving employment opportunities [7,8,23,24,25,26,27,28]. Moreover, in the context of internal displacement, migrants are either unable to or do not desire to cross international borders [29].

Once the threat to life and property has subsided, some of those internally displaced decide to return to their previous community [30,31]. Prior studies have identified major factors influencing *returnees* in the aftermath of a natural disaster, including the habitability of homes, access to affordable housing, financial burdens, the extent of restoration of public services and facilities, and a sense of place and identity [1,2,30]. Fear of future disasters, stress associated with recovery, and loss of employment are also influential [30]. In addition, education, employment, and other indicators of socioeconomic status may influence return migration in these circumstances [31].

Return migration may constitute an opportunity for internally displaced persons to reclaim what they lost and begin anew, but classic studies on the effects of natural disasters suggest that the process of starting over is difficult and life is never truly the same [32,33]. Returnees face multiple obstacles to reestablishing themselves successfully in their old communities [33], not the least of which is the continued perception of risk associated with the previous natural disaster [34]. Moreover, as Lori and Boyle [35] highlight, “a sudden influx of returnees can strain a country in the midst of rebuilding and has the potential to trigger additional problems for an already traumatized population” (p. 72). Given these challenges, it is plausible that return migration has strong effects on the health of returnees, yet return migration and health has received limited attention in policy and research [15].

### 1.2. Return Migration as a Determinant of Health

While there remains a paucity of studies examining return migration as a determinant of health [15], the burgeoning literature provides some guidance for the present study. It is anticipated that the overall health and well-being of returnees in the aftermath of a disaster is affected by cumulative exposure to social determinants of health and other risk factors associated with the disaster, the initial forced migration, the process of return, and circumstances following return [15,17]. As such, there is a multiplicity of critical junctures in a sequential pathway that may affect the health of returnees. For example, in a case/control study, researchers found that household ownership and use of bed nets was significantly lower in a displacement camp than in a nearby control village. This disparity in ownership and use of bed nets corresponded with a higher prevalence of malaria among children living in the displacement camp compared to the control village [36]. As such, exposure to these risk factors and diseases will continue to affect this displaced population even once they leave the displacement camp [15,36]. Given the multiple junctures that may affect the health of returnees, it is not surprising that health upon arrival varies substantially.

Some returnees arrive home to their previous communities healthy; e.g., those who were able to maintain employment in relocation destinations as well as access health and other public services [15]. Others are not so fortunate—migrants who were unemployed or received low wages; lived in dilapidated housing; had limited access to safe, nutritious food; and/or had difficulty accessing health care and other public services often return home less healthy than when they left [15,37]. Moreover, such populations, given their overall vulnerability, are likely to have been exposed to other risk factors such as poor sanitation that negatively affect health [15,36]. These returnees often have a wide range of health needs that require access to health care that may not exist or that they are unable to afford [15].

Often, internally displaced persons are forced to seek shelter in temporary resettlement camps; the living conditions in these settings often negatively influence the health status of returnees through increasing exposure to risk factors of chronic and/or infectious diseases as well as mental health conditions [15,36]. The negative impact of these exposures can persist even after returning to places of origin [15]. Moreover, it is often the case that the circumstances resulting in internal displacement also result in a breakdown of health services. In the wake of natural disasters, public health departments typically take on the role of maintaining the health of displaced persons [37]. However, these displaced persons often arrive with acute and chronic conditions [37]. When they arrive en masse, they often strain the already weakened public health services, which struggle to answer the increased demand [15,35]. In contrast, “returning to a supportive social network, language, and social norms and a health system that the returnee understands often contributes to improve the returnees’ physical, mental, and social well-being” [15] (p. 3).

### 1.3. Contextual Background: Mt. Merapi, Indonesia

Indonesia is a vast archipelago that includes approximately 17,500 islands, the largest of which include Java, Sumatra, Borneo, and Sulawesi. When both land and sea are included, Indonesia ranks geographically as the 7th largest country in the world, covering 1,919,440 square kilometers [38]. It is also home to approximately 252,000,000 people, making it the 4th largest country in population. Indonesia is also a nation of substantial linguistic and ethnic diversity, with over 700 spoken languages and 15 major ethnic groups that comprising 85% of the population. The largest of the major ethnic groups, the Javanese, make up roughly 40% of the total population [38].

**Figure 1:**
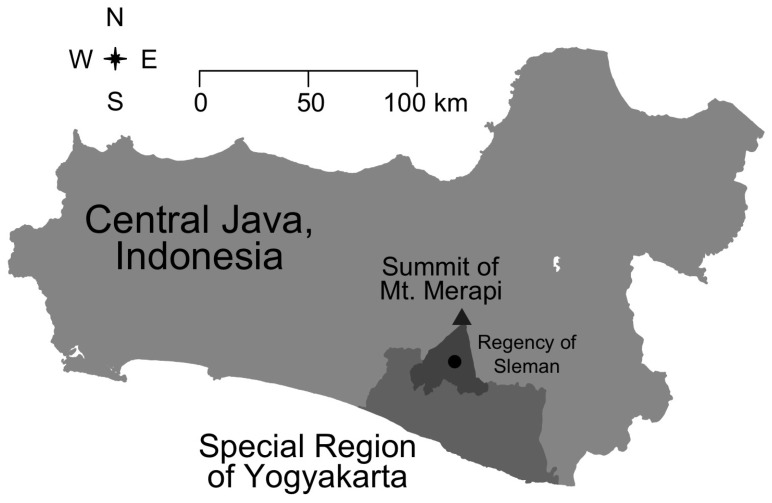
Mt. Merapi, Indonesia. Located in Central Java province and the Regency of Sleman within the Special Region of Yogyakarta.

Mt. Merapi is in the central region of Java, Indonesia; the most highly populated island on the planet, with an average population density of 1000 persons per square kilometer (see Figure 1). Of interest to this investigation, the population density within a 15-km radius of Mt. Merapi ranges from 0 to 5000 per square kilometer. Within this geographic area, roughly 98% of the population are ethnic Javanese [39]. ‘Sedumuk batuk senyari bumi’ is a common Javanese proverb, interpreted to mean that *‘dignity and land are things to strive for’*. The proverb highlights the attachment that Javanese people have to the land and to the community that they come from, which may help explain in part why they choose to return to Mt. Merapi despite the well-known risks [10,11].

In line with this, past studies have documented multiple areas in Indonesia where residents, despite well-documented risks and official relocation policies, are reluctant to resettle [9,12,40,41]. Of particular note to our study, Sontosudarmo [12] and Amin et al. [9] have showed that though threatened by the eruption of Merapi that takes place periodically every 4–5 years, residents choose to remain in the area. As Amin et al. [9] explains, residents “understand that disaster is something dangerous but an attempt to leave their place is not something they have to do because they have an attachment to Merapi as a place of origin, where they gain a sense of comfort and safety” (p. 35). Thus emerges a pattern of return migration in which displaced residents persist in returning to their communities and homes once the immediate risks of a disaster subside.

Recognizing that these migration patterns are likely associated with an increased risk of future disasters in the Mt. Merapi area, BNPB, in collaboration with other government agencies, endeavored to encourage the resettlement of risky residential areas for the purpose of hazard mitigation in the aftermath of the 2010 eruptions. To facilitate the resettlement process, a risk analysis was carried out to identify high risk areas. The assessment identified the southern slopes of Merapi as those at highest risk [10] and in line with these findings, the government sought to create incentives for villagers living along Merapi’s southern slopes to relocate to less hazardous areas through the use of various forms of recovery aid. The amount of assistance provided was based on the level of damages that an individual/household experienced in connection to the disaster; e.g., mild, moderate, or complete destruction. Those whose homes were destroyed were to be relocated and ultimately receive permanent housing. While waiting for the promised recovery assistance, people lived in temporary housing shelters. Ultimately, disaster victims were given a transition period of two years to stay in the temporary residence; after that, they would have to decide whether or not they were willing to relocate. In their research exploring possible connections between migration status and recovery aid in the aftermath of the 2010 eruptions, Muir et al. [13] report that the factors most strongly associated with continued displacement included the extent to which a household’s residence was destroyed and perceptions of the overall destruction that a household experienced due to the eruptions—findings that are consistent with other published studies on disasters and migration [1,2,30]. Alternatively, the provision of financial recovery aid was associated with increased probability of households having moved on to resettle in new communities located within the general area Mt. Merapi [13].

### 1.4. Summary and Expectations

Drawing upon the extant literature, we anticipate that returnees in the aftermath of a disaster are likely to report better health and well-being compared to those who have yet to leave displacement camps/shelters. However, within the context of this disaster, we also anticipate that those who have opted to move on rather than move back home may have superior health and well-being compared to those who have yet to leave displacement camps/shelters and those who have already returned home. Given the multiplicity of critical junctures that may affect the health of returnees described above, we further anticipate that these associations are subject to confounding and/or effect modification by other factors such as level of destruction experienced by the individual and/or their household, continued psychological impacts such as fear in relation to the disaster, and interventions by either government or NGOs to help migrants rebuild their lives and communities.

Given the lack of research on the impact of return migration on health in the aftermath of disasters, and the underlying proposition that it may be better to move on than to move back home, we propose the following research question: In the aftermath of a disaster, is it better to move home or move on? Which of these options results in superior improvements to health and/or well-being? Hereafter, we will examine the association between migration status and mental health while adjusting for factors that the extant literature suggests may confound this association. Below we detail the analytic methods we used to test these expectations in the aftermath of the 2010 Mt. Merapi volcanic eruptions.

## 2. Materials and Methods

To address this research question, we draw upon data from the “Community Recovery after a Natural Disaster: A Survey of Communities Affected by Mt. Merapi Eruptions” study. Research protocols and procedures used in this exploratory study were reviewed and approved, on 28 February 2012, by the Institutional Review Board for Human Subjects (IRB) at Brigham Young University. The survey questionnaire used in the study was developed in an iterative process by a research team including members from Indonesia and the United States. After initial development in English, the questionnaire was translated into Bahasa Indonesia by a translation team made up of research team members who were native speakers of either Bahasa Indonesia or English, but who were also fluent in their non-native language of either Bahasa Indonesia or English. The translation process included standard translation/back-translation steps in an effort to increase the accuracy and cultural appropriateness of the questionnaire. The survey instrument was used to structure a guided survey interview conducted between each respondent and interviewer. The data were collected by faculty and student research assistants at the Institute of Community Development Research Center in Yogyakarta, Indonesia. All interactions between the researchers and respondents were carried out in Bahasa Indonesia, and data were then translated into English and entered into a database for further statistical analysis.

The study was conducted 16 months after the 2010 eruptions. It was organized as a cross-sectional pilot study that included retrospective questions to document in detail the experiences of victims of the disaster, including their experiences related to disaster preparedness, mitigation, and recovery, as well as their overall experience of the emergency. This has strong implications for the overall power, or limitations, of the data collected to investigate migration in response to the eruptions.

### 2.1. Sampling Procedures

**Figure 2:**
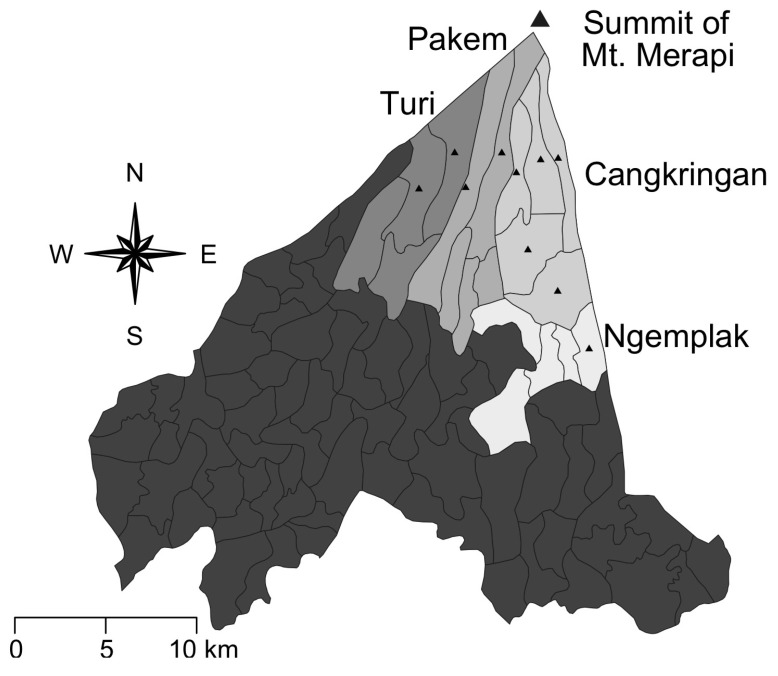
Regency of Sleman.

Respondent identification was conducted using stratified multistage randomized cluster sampling to achieve two specific goals. First, to generate a sample representative of varying levels of destruction caused by the eruptions. Second, to generate a sample of respondents that included those who were still living in a disaster shelter, those who had returned to their previous community, and those who had moved on to a new community. The Merapi eruptions struck five Regencies around the mountain: Boyolali, Klaten, Magelang, and Muntilan located within Central Java Province and Sleman Regency located within the Special Region of Yogyakarta (See Figure 1). The damages caused by the disaster varied from one region to another. After taking into consideration time, costs, distance, previously identified patterns of return migration [9,10,11,12], and that this would be a pilot project on disaster mitigation, ten villages in Sleman Regency were chosen as the study location (See Figure 2). Those ten villages are spread across 4 districts: Turi, Ngemplak, Cangkringan, and Pakem.

The ten villages were selected based on the relative impact the Merapi eruptions had on each village, which ranged from those most severely affected to those only slightly affected. The villages classified as severely damages were Girikerto Villages (District of Turi), Hargobinangun Village (District of Pakem), Umbulharjo, Kepuharjo, Glagahharjo, Argomulyo Villages (District of Cangkringan) and Sindumartani Village (District of Ngemplak). The villages considered to be slightly affected were Wonokerto Village (District of Turi), Purwobinangun Village (District of Pakem), and the village of Wukirsari (District of Cangkringan). Within these ten villages, data collection was further stratified into a total of 40 Hamlets or Sub-Villages (Dusun/Padukuhan).

The districts of Turi, Pakem, and Cangkringan are located near the summit of Mt. Merapi. Conversely, Ngemplak is located to the south of Cangkringan District in the eastern area of Sleman Regency. In this district, most victims were those who lived near a river and the level of destruction that they experienced varied; i.e., some people were completely unaffected, others had their homes damaged minimally, and for others their homes and livelihoods were destroyed completely. The extent of the devastation in some locations was such that entire villages vanished, leaving only what appeared to be a field of sand where they once stood.

The process for selecting respondent village or shelter communities included several steps. First, a district was divided along its radius from north to south and from east to west. Girikerto and Wonokerto were the villages closest to the peak of Mt. Merapi in the Turi District. In the Pakem District, Turgo was the village closest to the peak of the volcano. In Cangkringan, Kinahrejo was the northernmost village with the closest proximity to the volcano. Finally, in the Ngemplak District, Sindumartani was the village damaged most severely. The remainder of the villages selected for inclusion in the study experienced damage that ranged from moderate to slight.

### 2.2. Respondent Selection

**Figure 3:**
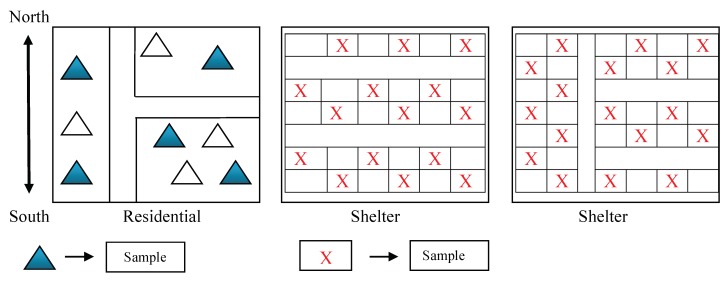
Respondent Selection Schematic. The 400 households originally surveyed were identified using stratified multistage randomized cluster sampling.

Within the study sites, respondent selection was organized to obtain a sample that included respondents who were still displaced and living in a shelter, respondents who had returned to their previous communities, and respondents who had moved on to settle in new communities. The method for respondent selection was similar to the selection process used to identify villages and shelters within the districts; i.e., residence selection started from the northernmost part of a village or shelter community and then moved from east to west and while also gradually moving south (See Figure 3). Within this process, residences were chosen randomly. Rather than operating with a specified sample frame, respondent selection was carried out to obtain a specified number of responses from each of the identified villages. Forty respondents were selected from each village, with one respondent per household. With 10 different villages or shelter communities included in our sample frame, we obtained a total respondent sample of 400 (two respondents were removed from the analytic sample because they were under the age of 18).

**Figure 4:**
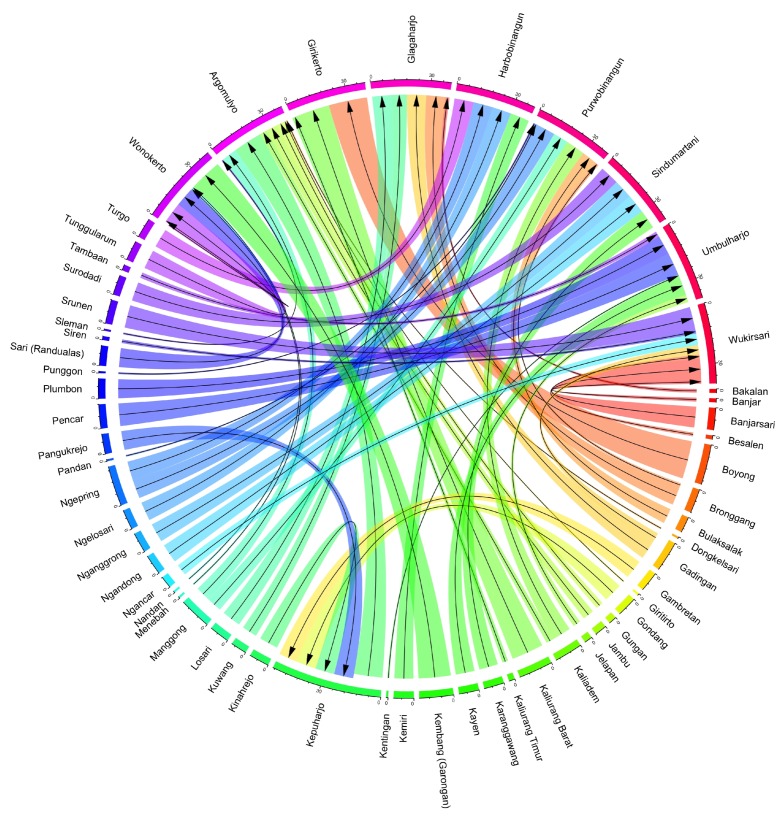
Migrations Flows from Sending to Receiving Villages. The 398 respondents in our survey reported coming from 52 villages of origin or sending villages prior to the evacuation order that corresponded with the volcanic eruptions. The respondents were identified through our sampling procedure which first selected the 10 receiving villages,

It is important to distinguish between the villages that respondents were sampled from and their villages or towns of origin (e.g., their “sending” village). The 398 respondents ultimately included in our analyses originated from 50 different villages or towns (See Figure 4). There is some overlap between the villages used for sampling and the villages of origin due to the fact that a portion of the sample had returned to their original village at the time of the survey, but many were still displaced or had moved on to new locations. The distribution of respondents from these various villages was such that roughly a third of the sending villages were represented by only one person. In contrast, many of the sending villages were represented by at least 10 respondents and several villages had 15–20.

The sample procedures and protocol for respondent selection were established with the goal of creating a sample representative of the varying levels of destruction that respondents experienced from the eruption. However, as a post-disaster study, we are unable to directly assess the extent to which our sample is representative of the pre-disaster population. Some of the persons displaced by the eruption may have migrated beyond the geographic scope of our study. While this is a concern, qualitative data gathered through interviews and focus groups suggest that the vast majority of displaced persons remained within the geographic region surrounding Mt. Merapi, a pattern consistent with research carried out by the Indonesian disaster management agency BNPB [10] and documented in related studies [11]. In addition, comparing demographic characteristics of our sample with 2010 census data for Indonesia [42] suggests that the distribution of respondents within our sample is comparable to that in the Special Region of Yogyakarta and similar to the general population of Indonesia (see Appendix A). For example, our study sample almost matches the population distribution of DI Yogyakarta in terms of religion and is comparable to that for Indonesia, overall. While our study sample had a somewhat higher distribution of educational attainment in comparison to the population of DI Yogyakarta, this differences is likely attributable to the age distributions in our sample as educational attainment in DI Yogyakarta decreases with age, so a younger sample in our data would result in somewhat elevated percentages for education attainment.

### 2.3. Measures

The dependent variable used in the analysis measured self-reported mental health. The original question in the guided interview survey instrument stated, “Ask them to rate their mental health on a scale from 1 to 5, with 1 being very good and 5 being very poor”. For the purpose of this study, the original categories of this variable were collapsed and re-coded such that the new variable was coded as an ordinal variable with 1 = Poor or Very Poor, 2 = Average, and 3 = Good or Very Good. This was done to facilitate analysis given the relatively small sample size collected.

Independent variables were divided into four sets. First, the primary variable of interest captured *Migration Status* in terms of whether or not a respondent and/or their household were displaced, in transition, had moved on, or had moved home (100% of our sample was originally displaced due to an evacuation order). The variable was constructed from responses to two questions in the survey: (1) Have they [the respondent] returned to their previous community since the disaster? (2) Do they [the respondent] currently live in temporary housing? (shelter, barrack, government relocation site, etc.)? Using responses to these questions, the following categories were constructed: (1) *Displaced*—respondents who indicated that they had yet to return to their previous community and were still living in temporary housing; (2) *Moved Home*—respondents who indicated that they had returned to their previous community and no longer living in temporary housing; (3) *In Transition*—respondents who indicated that they had returned to their previous community and were still living in temporary housing; (4) *Moved On*—respondents who indicated that they had yet to return to their previous community and no longer living in temporary housing. *Displaced* was set as the reference category.

The next group of variables include baseline demographic data. We measured *Age* as a continuous variable ranging from 18 to 82. Preliminary models also included a squared transformation of Age to assess whether any association with age was nonlinear; however, results from these analyses indicated that this was not statistically significant, and the variable was removed from subsequent analyses. *Sex* was included as a dichotomous variable with 1 = Male. *Marital Status* was also included as a categorical variable with 1 = Married (reference group), 2 = Not Married, 3 = Separated, 4 = Divorced, and 5 = Widowed. *Education* was measured on an ordinal scale ranging from 1 = No Education (reference group) to 4 = High School and Beyond (*High School Plus*). *Income* was measured as a categorical variable using the Indonesian Rupiah and coded as 1 = Less than 500,000 (reference group); 2 = 500,001 to 800,000; 3 = 800,001 to 1,000,000; and 4 = 1,000,000+. In addition, we included dummy variables to contextualize our results by geographic location. Specifically, dummy variables were included for the *Turi*, *Ngemplak*, and *Cangkringan* districts, while the *Pakem* district served as the comparison group.

A third set of independent variables assessed the level of destruction that respondents experienced as a result of the volcano. *Percentage of Personal Assets Lost* was measured as a continuous variable. *Fears Nature’s Wrath* was coded as a dichotomous variable (1 = Strongly Fears Nature’s Wrath).

The final set of independent variables assessed the extent to which recovery assistance was provided to the respondents. Specifically, dummy variables were created to assess if the respondent or anyone in his/her household received *Financial Recovery Aid*, *Housing Recovery Aid*, or *Food Recovery Aid* (coded as 1 = Received).

## 3. Analytic Strategy

To address our research questions, we assessed the probability of a respondent having reported good versus average or poor mental health by specifying ordered logistic regression models. Because this was an exploratory investigation, ordered logistic regression models were estimated first for each independent variable of interest individually to determine whether a statistically significant relationship existed. These factors were then evaluated in unison in a series of regression models. Ultimately, two models were selected as a summary presentation of our findings.

**Model 1** assessed the relationship between *Mental Health* and *Migration Status* while adjusting for *Age*; *Marital Status*; *Education*; *Income*; *Geographic District*; *Financial Recovery aid*, *Housing Recovery aid*, and *Food Recovery aid*; and *Fear of Nature*.

**Model 2** assessed the relationship between *Mental Health* and *Migration Status* while adjusting the control variables included in Model 1 as well as for assets lost during the disaster.

## 4. Results

The descriptive statistics in Table 1 present several preliminary findings worth highlighting. Concerning the overall migration status at the time of the survey, the prevalence of return migration among the 398 respondents in the sample was roughly 52%, with 206 respondents having returned to their prior community and living in permanent housing. Another 69 (17.3%) respondents were “in transition” as they had also returned to their original community, but were still living in temporary housing at the time of the survey. Only 22 (5.5%) respondents indicated that they had “moved on” to resettle in a new community. This contrasts with the 101 (25.4%) of respondents still displaced and living in temporary relief shelters at the time of the survey. These distributions are consistent with the aforementioned tendency for individuals/households living near Mt. Merapi to return to their original communities after eruptions, despite their awareness that Mt. Merapi is an active volcano that erupts regularly [9,10,11,12]. With regards to the breakdown of migration status by groupings of mental health status, the largest contrast between the general trend and specific groupings is seen for those reporting poor mental health; e.g., 50% of respondents reporting poor mental health were displaced at the time of the survey, this is a rather large contrast to the 25% displaced in the overall sample. Likewise, a smaller percentage of respondents reporting poor mental health had either “moved home”, “moved on”, or were “in transition” at the time of the survey compared to the overall sample. These patterns suggest a possible association between migration outcomes in the aftermath of the disaster and the mental health status of respondents at the time of the survey.

In terms of demographic characteristics, there was a slight majority (60.8%) of male respondents. The distribution of respondent sex among the different groupings of mental health status varied somewhat, suggesting a possible association between respondent sex and mental health status. In contrast, the average age of respondents in the study sample was 46.5 years, which was fairly consistent across the different groupings of mental health status. Likewise, the vast majority of respondents were married at the time of the survey, and this distribution held for the different groupings of mental health status. The vast majority of respondents had completed at least a junior high level (26.9%) or high school level (38.7%) of education, with another 29.6% completing at least primary education. The patterning of education attainment within the different groupings of mental health status shown in Table 1 suggests that higher levels of education may be associated with improved mental health status in the aftermath of a disaster.

With regards to the extent of destruction experienced and the possible impact of recovery aid, the average loss of personal assets reported by respondents was 36.5%. This distribution of assets lost varied substantially across the different groupings of mental health status; e.g., the average proportion of personal assets lost by those reporting good mental health was only 30.5%. In contrast, those reporting average mental health reported a mean a loss of 44.2% of personal assets and respondents reporting poor mental health reported an average a loss of 62.0% of personal assets, suggesting that there was a persisting association between assets lost during the disaster and the mental health status of respondents at the time of the survey. In terms of lingering perceptions of the disaster, 272 (68.3%) respondents reported that they still strongly feared the wrath of nature at the time of the survey. Finally, at the time of data collection, 183 of the 398 respondents reported that they had received financial recovery aid, 254 of the 398 respondents reported that they had received food recovery aid, and 46 of the 398 respondents reported that they had received housing recovery aid.

Results (Tables were organized using Stargazer [43] in *R* [44]) from the ordinal logistic regression models are displayed as adjusted proportional odds ratios [45] with 95% confidence intervals in Table 2.

**Figure 5:**
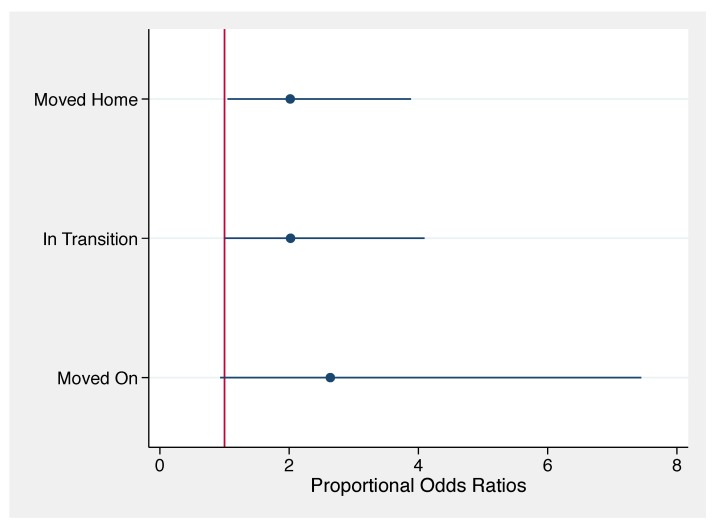
Adjusted Proportional Odds Ratios for Associations between Self-Reported Mental Health and Migration Status from Model 1 in Table 2. Respondents who had either moved on, were in transition, or moved home were much less likely to report either average or poor mental health compared to displaced respondents (reference category).

Compared to respondents who were still displaced at the time of the survey, respondents who had “moved home” or were “in transition” were proportionally more likely to report either good mental health as opposed to poor or average mental health after adjusting for a variety of demographic and disaster related variables; i.e., the POR for “moved home” and “in transition” were POR = 2.02 [95% CI = 1.05, 3.91] and POR = 2.02 [95% CI = 1.00, 4.14] respectively. Likewise, respondents who had “moved on” were proportionally more likely to report good mental health rather than either average or poor mental health (POR = 2.64 [95% CI = 0.96, 7.77] with a corresponding *p*-value of 0.067) compared to respondents who were still displaced at the time of the survey. These associations are visually summarized in Figure 5, which emphasizes the protective effect of transitioning beyond displacement suggested by our results juxtapose the rather large confidence intervals associated with these results. Apart from migration status, it is worth noting that education attainment was also associated with mental health status. Compared to respondents with no education, respondents were more likely to report good mental health in the aftermath of the disaster if they had attained either primary (POR = 5.78 [95% CI = 2.14, 15.78]), junior high (POR = 3.83 [95% CI = 1.40, 10.56]), or “high school plus” (POR = 4.09 [95% CI = 1.53, 11.02]) levels of education.

**Figure 6:**
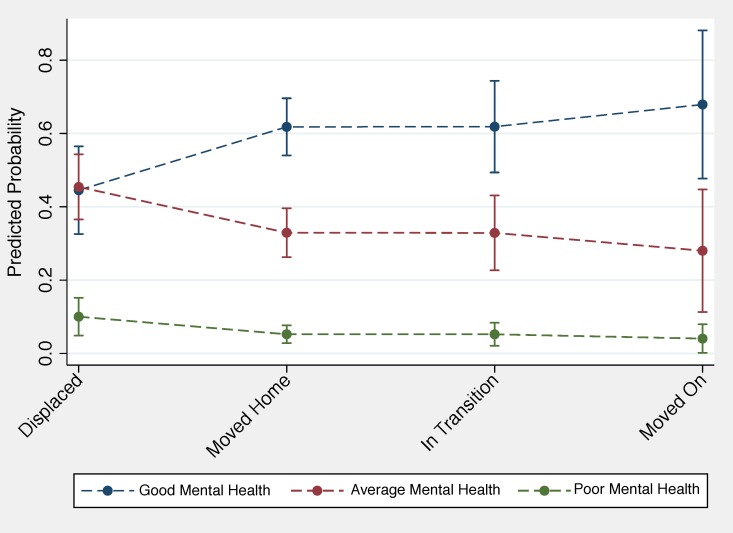
Adjusted Predicting Probability of Good, Average, or Poor Mental Health by Respondent Migration Status. Respondents who had moved on were much more likely to report having good mental health while those who had moved home or were still in transition were only marginally more likely to report good mental health compared to displaced respondents after adjusting for additional control variables. In contrast, respondents who had moved on were much less likely to report having average or poor mental health while those who had moved home or were still in transition were only marginally less likely to report average or poor mental health compared to displaced respondents after adjusting for additional control variables.

In an attempt to render these results more intuitive than a table of coefficients or odds ratios, predicted probabilities were estimated and organized into a data visualization in Figure 6, which include 95% confidence intervals and represent the associations between migration status and the probability of having good, average, or poor mental health. While maintaining the same general pattern, the predicted probabilities depict a more nuanced association between migration status and mental health status than that conveyed by the odds ratios. The strongest distinctions between the different migration outcomes are seen between respondents who reported good versus respondents who reported only average mental health—the probability for reporting poor mental health was low across all migration outcomes with minimal variation. There is a clear positive association between respondents reporting good mental health and reporting that they had either “moved home” or were “in transition” compared to still living in a displacement shelter. The respondents who had the highest probability of reporting good mental health were those who had “moved on”. In contrast, the highest predicted probability for reporting having only average mental health were for those respondents who were still displaced at the time of the survey. The predicted probabilities for respondents who reported that they had either “moved home” or were “in transition” were markedly lower, and the lowest predicted probability for average mental health were for those respondents who had “moved on”. A similar, though much less distinct pattern is seen for respondents who reported poor mental health. The highest predicted probability for reporting poor mental health were for those respondents who were still displaced at the time of the survey; in contrast, the lowest predicted probability for reporting poor mental health were for those respondents who had “moved on”.

While the associations between migration status and mental health status are of substantial strength and statistically significant for respondent who had either “moved home” or were “in transition”, the relative strength and statistical significance of these associations diminished after adjusting for the association between mental health and percentage of personal assets lost during the disaster (POR = 0.99 [95% CI = 0.98, 0.99]) (see Model 2 in Table 2). Given prior findings suggesting that the level of destruction experienced during the disaster was associated with subsequent migration outcomes [13,14], these findings may suggest a possible mediation/modification effect in which the association between the level of destruction a household experienced during the disaster and subsequent mental health outcomes are either mediated or moderated by migration decisions. In contrast, the protective effect of education is robust to inclusion of this additional variable.

## 5. Discussion

In this study, we set out to explore the extent to which various migration outcomes were associated with superior mental health status in the aftermath of the 2010 Mt. Merapi volcanic eruptions. With this inquiry as the guiding premise, we explored whether “moving home” or “moving on” resulted in reports of superior mental health compared to remaining displaced in resettlement shelters. Our results suggest that in the wake of a disaster, any improvement in migration status beyond remaining displaced is likely to improve mental health; yet, at least within the context of this disaster, it may have been best to “move on”. Respondents who had either returned to their original community or had moved on to settle in a new one were both more likely to indicate positive health compared to respondents still living in temporary relief shelters; however, respondents who had moved on to settle in a new community were more likely to report better health overall. These results are consistent with the proposed hypothesis that perhaps it is better to move on than to move home. However, the results for respondents who had moved on were only marginally significant (*i.e., given the corresponding p-value, we can only be 93.3% confident in our results as opposed to the standard 95%*), likely the result of having only a relatively small number of respondents reporting that they had moved on at the time of the study—a limitation that should be addressed in a follow-up study in the Mt. Merapi area.

Our results are likewise consistent with findings in the disaster literature that suggest that the overall health and well-being of returnees in the aftermath of a disaster is affected by cumulative exposure to social determinants of health and other risk factors associated with the disaster, the initial forced migration, the process of return, and circumstances following return [15,17]; e.g., with regards to our results, the influence of migration status was significant up until taking into account the proportion of personal assets that respondents estimated were lost as a result of the disaster, which was strongly associated with poorer mental health. For instance, internally displaced persons are often forced to seek shelter in temporary resettlement camps; such was the case for the entirety of the households surveyed in our study as 100% were originally displaced due to the eruptions. The living conditions in such settings often negatively influence the health status of returnees through increasing exposure to risk factors of chronic and/or infectious diseases as well as mental health conditions [15,36]. Our finding that poor mental health was most strongly associated with those respondents who remained displaced is consistent with the broader literature. In contrast, moving forward, both figuratively and literally, by leaving the resettlement camps is often associated with improvements in physical and mental health as well as social well-being [15].

While this study contributes to the literature on return migration as a determinant of health in the aftermath of a natural disaster, it is not without limitations. The most significant limitation is that these data were part of a cross-sectional pilot study organized to document in detail the experiences of victims of the disaster. This has several ramifications. First, there is need for caution in interpreting our results as they are associations and our ability to make causal inference is limited; i.e., our interpretation that these associations suggest possible treatment effects is based primarily upon the temporal sequence of events assessed through the use of retrospective questions. Second, the ability to leverage these data for statistical analyses investigating migration status was constrained. Investigating migration outcomes was not the main purpose of the data; as a result, more targeted questions related to migration were not included in the survey—a limitation that should be addressed in future research in the Mt. Merapi area. Of particular concern is the paucity of temporally related variables. More robust and detailed analyses would be possible if a larger number of the variables had considered time with retrospective components, or, more ideally, if a baseline study was already in place.

Furthermore, our reliance upon self-reported measures of health is not ideal—future data collection should consider alternative means of assessing the mental health of participants and/or other measures of vulnerability or resilience. We further note that only a small proportion of respondents indicated that they had poor mental health in this pilot study, even in the wake of having been displaced and having experienced a variety of losses related to the disaster. It is possible that obtaining a larger sample size in a follow-up study may help achieve a more normally shaped distribution. Alternatively, the small proportion of respondents reporting poor mental health may be partially attributable to response bias. Despite efforts to prepare a survey instrument that was linguistically and culturally appropriate and accurate for the study setting, the small proportion of respondents reporting poor mental health may be the result of measurement error steaming from misclassification bias. To the extent that potential bias occurred non-differentially, it would result in more conservative estimates of the overall effect of migration status on mental health. With this in mind, future research should seek to attenuate the potential for response bias and/or misclassification bias by using superior metrics for assessing the mental health of respondents.

In addition, as a pilot study, only a relatively small sample of data was collected. This limits the statistical power of our analyses; e.g., we have a small sample size for the “poor mental health” category in our dependent variable even after collapsing the “poor” and “very poor” categories. It may be that some indicators, including our explanatory variable of interest, may have had stronger statistical significance if the study size was larger. On a practical level, while the findings presented herein are substantively interesting, it would be hard to strongly argue for policy changes based upon the experiences of the 22 participants who had “moved on” at the time of the survey. It is perhaps fortuitous that our results are consistent with the current local policies seeking to create incentives for households to move on and resettle in less hazardous areas. With these limitations in mind and given the regularity with which Mt. Merapi erupts, future research should include the formal establishment of a research site around Mt. Merapi to facilitate additional data collection. This data collection should aim to expand the current sample size and thereby improve statistical power while also allowing for the collection of longitudinal data to provide more robust findings to inform policy decisions.

An additional limitation is related to the potential for bias within our data. Sample bias may exist as our sample frame only included the geographic region surrounding Mt. Merapi. It is possible that some of the persons displaced by the eruption migrated beyond the geographic scope of our study and it is difficult to assess how this potential sample bias may affect our results. In addition, the timing of the data collection may have occurred too soon after the eruption. This could result in failing to observe alternative trends that would have emerged if the data were collected at a more distant time relative to the disaster. As previously stated, a straightforward solution to this limitation would be to conduct a follow-up study in the near future or to establish a permanent research site to collect data over time and whereby households could be tracked down, even if they move out of the immediate area.

Finally, as with the majority of natural disasters, these findings come from a specific event, occurring at a specific time and in a specific location. This is important given that the destruction resulting from Mt. Merapi’s eruption only became a natural disaster through interaction with local social systems. As Perry [46] states, “It is not the hurricane wind or storm surge that makes the disaster; these are the sources of the damage. The disaster is the impact on individual coping patterns and the inputs and outputs of social systems” (12). In as much as a disaster is a social phenomenon that is inherently local [46,47], it is difficult to gauge the extent to which our findings are generalizable beyond the context of Java, Indonesia. At a minimum, these findings could inform other research studies through incorporation into a systematic review or meta-analysis of indicators of return migration in the aftermath of natural disasters. Despite these limitations, this study contributes meaningful insights regarding the role of return migration as a determinant of health in the aftermath of a natural disaster.

Despite these limitations, we anticipate that these exploratory findings will be of interest to local policy makers and also contribute to the ongoing discussion in the broader scholarly community investigating migration as a social determinant of mental health in the context of disasters (These results will certainly inform our own future investigations into how these regular eruptions affect the lives of those living on and around Mt. Merapi). As discussed in this study, return migration may constitute an opportunity for migrants to reclaim what they lost and begin anew in the aftermath of natural disasters, but classic studies on the effects of disasters suggest that the process of starting over is difficult and life is never truly the same [32,33]. Thus, the trip home is but the first step in regaining what was lost, and therefore, return may not be the best choice. While buildings can be rebuilt, the same is not always true of the past, and the future is likewise uncertain. Given the findings in this study, for some victims of disasters it might be better to move on rather than move back—especially in a world where disasters are an increasingly frequent reality.

## Figures and Tables

**Table 1:** Descriptive Statistics by Self-Reported Mental Health Status.

Categorical Variables		Good	Average	Poor	Overall
**n**		**230**	**140**	**28**	**398**
**Migration Status**					
Displaced		54 (23.5)	33 (23.6)	14 (50.0)	101 (25.4)
Moved Home		121 (52.6)	75 (53.6)	10 (35.7)	206 (51.8)
In Transition		41 (17.8)	25 (17.9)	3 (10.7)	69 (17.3)
Moved On		14 (6.1)	7 (5.0)	1 ( 3.6)	22 (5.5)
**Sex**					
Female		85 (37.0)	58 (41.4)	13 (46.4)	156 (39.2)
Male		145 (63.0)	82 (58.6)	15 (53.6)	242 (60.8)
**Marital Status**					
Married		204 (88.7)	124 (88.6)	26 (92.9)	354 (88.9)
Not Married		9 (3.9)	5 (3.6)	0 ( 0.0)	14 (3.5)
Separated		0 (0.0)	3 (2.1)	0 ( 0.0)	3 (0.8)
Divorced		3 (1.3)	2 (1.4)	0 ( 0.0)	5 (1.3)
Widowed		14 (6.1)	6 (4.3)	2 ( 7.1)	22 (5.5)
**Education**					
None		5 (2.2)	10 (7.1)	4 (14.3)	19 (4.8)
Primary		75 (32.6)	39 (27.9)	4 (14.3)	118 (29.6)
Junior High		61 (26.5)	35 (25.0)	11 (39.3)	107 (26.9)
High School+		89 (38.7)	56 (40.0)	9 (32.1)	154 (38.7)
**Income**					
0–500,000		65 (28.3)	33 (23.6)	11 (39.3)	109 (27.4)
500,001–800,000		60 (26.1)	41 (29.3)	7 (25.0)	108 (27.1)
800,001–1,000,000		57 (24.8)	29 (20.7)	4 (14.3)	90 (22.6)
1,000,000+		48 (20.9)	37 (26.4)	6 (21.4)	91 (22.9)
**District**					
Pakem		117 (50.9)	62 (44.3)	20 (71.4)	199 (50.0)
Turi		47 (20.4)	29 (20.7)	4 (14.3)	80 (20.1)
Ngemplak		23 (10.0)	17 (12.1)	0 (0.0)	40 (10.1)
Cangkringan		43 (18.7)	32 (22.9)	4 (14.3)	79 (19.8)
**Fears Nature’s Wrath**					
No		69 (30.0)	47 (33.6)	10 (35.7)	126 (31.7)
Yes		161 (70.0)	93 (66.4)	18 (64.3)	272 (68.3)
**Financial Aid**					
Not Received		129 (56.1)	79 (56.4)	7 (25.0)	215 (54.0)
Received		101 (43.9)	61 (43.6)	21 (75.0)	183 (46.0)
**Housing Aid**					
Not Received		204 (88.7)	121 (86.4)	27 (96.4)	352 (88.4)
Received		26 (11.3)	19 (13.6)	1 ( 3.6)	46 (11.6)
**Food Aid**					
Not Received		85 (37.0)	49 (35.0)	10 (35.7)	144 (36.2)
Received		145 (63.0)	91 (65.0)	18 (64.3)	254 (63.8)
Continuous Variables		Good	Average	Poor	Overall
**Age** (mean (SD))		45.89 (14.21)	47.69 (14.48)	46.00 (13.26)	46.53 (14.23)
**Percent of Personal Assets Lost** (mean (SD))		30.50 (39.31)	41.30 (44.82)	62.00 (42.23)	36.51 (42.32)

**Table 2:** Adjusted Ordinal Logistic Regression Results Evaluating Mental Health by Migration Status.

	*Dependent Variable:* Poor, Average, or Good Mental Health	
	(Model 1)	(Model 2)
**Migration Status**	
Displaced	1.00 (1.00, 1.00)	1.00 (1.00, 1.00)
Moved Home	2.02 * (1.05, 3.91)	1.28 (0.63, 2.60)
In Transition	2.02 * (1.00, 4.14)	1.97 (0.97, 4.06)
Moved On	2.64 (0.96, 7.77)	1.70 (0.60, 5.17)
**Age**	0.99 (0.97, 1.00)	0.98 (0.97, 1.00)
**Marital Status**	
Married	1.00 (1.00, 1.00)	1.00 (1.00, 1.00)
Not Married	0.98 (0.32, 3.42)	0.95 (0.30, 3.37)
Separated	0.11 * (0.01, 0.85)	0.09 * (0.01, 0.76)
Widowed	1.37 (0.53, 3.84)	1.20 (0.45, 3.46)
Divorced	1.55 (0.25, 13.10)	1.72 (0.27, 14.65)
**Sex**	
Female	1.00 (1.00, 1.00)	1.00 (1.00, 1.00)
Male	1.52 (0.97, 2.41)	1.64 * (1.03, 2.62)
**Education**	
None	1.00 (1.00, 1.00)	1.00 (1.00, 1.00)
Primary	5.78 *** (2.14, 15.78)	5.85 *** (2.11, 16.40)
Junior High	3.83 ** (1.40, 10.56)	4.27 ** (1.52, 12.13))
High School+	4.09 ** (1.53, 11.02)	4.33 ** (1.58, 11.98)
**Income**	
Less than 500,001 RP	1.00 (1.00, 1.00)	1.00 (1.00, 1.00)
500,001–800,000 RP	0.90 (0.51, 1.57)	0.95 (0.54, 1.68)
800,001–1,000,000 RP	1.30 (0.71, 2.41)	1.31 (0.71, 2.45)
1,000,000+ RP	0.64 (0.34, 1.19)	0.64 (0.34, 1.21)
**Districts**	
Pakem	1.00 (1.00, 1.00)	1.00 (1.00, 1.00)
Turi	0.69 (0.35, 1.35)	0.67 (0.33, 1.33))
Ngemplak	1.40 (0.65, 3.10)	1.18 (0.54, 2.67)
Cangkringan	0.58 (0.28, 1.21)	0.44 * (0.21, 0.94))
**Financial Aid**
Not Received	1.00 (1.00, 1.00)	1.00 (1.00, 1.00)
Received	0.64 (0.38, 1.06)	0.57 * (0.34, 0.95))
**Housing Aid**
Not Received	1.00 (1.00, 1.00)	1.00 (1.00, 1.00)
Received	1.10 (0.54, 2.28)	1.04 (0.50, 2.18)
**Food Aid**
Not Received	1.00 (1.00, 1.00)	1.00 (1.00, 1.00)
Received	1.12 (0.69, 1.82)	1.16 (0.71, 1.90)
**Fears Nature’s Wrath**	1.30 (0.81, 2.09)	1.35 (0.84, 2.18)
**Percent Personal Assets Lost**		0.99 *** (0.98, 0.99)
Observations	398	398

*Note:* * p< 0.05; ** p< 0.01; *** p< 0.001.

## Appendix A

**Table A1:** Statistics for Comparing Sample to General Population.

	Study Sample %	Special Region of Yogyakarta %	Indonesia %
**Demographic Characteristics:**			
Marital Status			
Single	3.5	32.6	31.9
Married	89.0	59.0	60.5
Divorced	2.0	1.4	1.8
Widowed	5.5	6.8	5.5
Religion			
Islam	92.2	92.0	87.2
Christian	7.5	7.5	9.8
Other	0.3	0.5	3.0
Education Attainment			
None	5.0	10.0	21.8
Some Primary	29.6	36.5	28.5
Lower Secondary	26.7	16.5	20.2
Upper Secondary and Beyond	38.7	37.0	29.5

Note: Columns may not sum to 100% due to rounding.

Comparison data obtained from Indonesia’s 2010 census [42], The marital status of the study sample is comparable to percentages reported for DI Yogyakarta and Indonesia overall in terms of divorced and widowed. The higher proportion of married compared to single is likely attributable to respondent status as head of household. In terms of religion, our study sample almost matches the population distribution DI Yogyakarta and is comparable to that for Indonesia, overall. Our study sample has a somewhat higher distribution of educational attainment in comparison to the population of DI Yogyakarta, an area that is known for having higher education attainment compared to national averages [48]. This difference is likely attributable to the age distributions in our sample as educational attainment in DI Yogyakarta decreases with age, so a younger sample in our data would result in somewhat elevated percentages for education attainment.
